# Comparison of Clinical Efficacy and Safety of Metformin Sustained-Release Tablet (II) (Dulening) and Metformin Tablet (Glucophage) in Treatment of Type 2 Diabetes Mellitus

**DOI:** 10.3389/fendo.2021.712200

**Published:** 2021-09-30

**Authors:** Li-xin Guo, Guo-en Liu, Li Chen, Hai-fang Wang, Jian Guo, Xian-ling Zheng, Bin-hong Duan, De-zhong Wang, Wei Zhu, Kun Wang, Wan-shou Tan, Qiu Chen, Quan-zhong Li, Jing Yang, Qiu Zhang, Pei-feng Xie, Min-xiang Lei

**Affiliations:** ^1^ Department of Endocrinology, Beijing Hospital, National Center of Gerontology, Institute of Geriatric Medicine, Chinese Academy of Medical Science, Beijing, China; ^2^ National School of Development, Peking University, Beijing, China; ^3^ Department of Endocrinology, Qilu Hospital of Shandong University, Jinan, China; ^4^ Department of Endocrinology, The First Hospital of Handan City, Handan, China; ^5^ Department of Endocrinology, Tianjin Hospital of Integrated Traditional Chinese and Western Medicine (ITCWM) Nankai Hospital, Tianjin, China; ^6^ Department of Endocrinology, Handan Central Hospital, Handan, China; ^7^ Department of Endocrinology, Heilongjiang Provincial Hospital, Harbin, China; ^8^ Department of Endocrinology, Tengzhou Central People’s Hospital, Tengzhou, China; ^9^ Department of Endocrinology, Beijing Aerospace General Hospital, Beijing, China; ^10^ Department of Endocrinology, Nanjing Jiangning Hospital, Nanjing, China; ^11^ Department of Endocrinology, The Central Hospital of Shaoyang, Shaoyang, China; ^12^ Department of Endocrinology, Affiliated Hospital of Chengdu University of Traditional Chinese Medicine, Chengdu, China; ^13^ Department of Endocrinology, Henan Provincial People’s Hospital, Zhengzhou, China; ^14^ Department of Endocrinology, First Hospital of Shanxi Medical University, Taiyuan, China; ^15^ Department of Endocrinology, First Affiliated Hospital of Anhui Medical University, Hefei, China; ^16^ Department of Endocrinology, Dongfang Hospital of Beijing University of Chinese Medicine, Beijing, China; ^17^ Department of Endocrinology, Xiangya Hospital Central South University, Changsha, China

**Keywords:** type 2 diabetes mellitus, metformin hydrochloride sustained-release, Dulening, Glucophage, diabetes

## Abstract

**Objectives:**

This study investigated the clinical efficacy and safety of metformin hydrochloride sustained-release (SR) tablet (II) produced by Dulening and the original metformin hydrochloride tablet produced by Glucophage in the treatment of type 2 diabetes mellitus (T2DM).

**Methods:**

This randomized, open and parallel controlled clinical trial consecutively recruited a total of 886 patients with T2DM in 40 clinical centers between May 2016 and December 2018. These patients were randomly assigned to the Dulening group (n=446), in which patients were treated with Dulening metformin SR tablets, and the Glucophage group (n=440), in which patients were treated with Glucophage metformin tablets, for 16 weeks. The changes in the levels of glycated hemoglobin (HbAc1) and fasting blood glucose (FBG) as well as weight loss were compared between these two groups. Also, the overall incidence of adverse drug reactions (ADRs) and the incidence of ADR of the gastrointestinal system observed in patients of these two groups were also compared.

**Results:**

There were no significant differences in demographic and basal clinical characteristics between these two groups. The Dulening and Glucophage groups showed comparable levels of decrease in HbA1c levels, FBG and weight loss after 12-week treatment (all p>0.05). The Dulening group had a significantly lower overall incidence of ADRs as well as gastrointestinal ADR than the Glucophage group.

**Conclusions:**

Metformin SR tablets (II) and the original metformin tablets exhibit similar therapeutic efficacy in the treatment of T2DM, but metformin SR tablets (II) has the significantly lower incidence of ADRs than the original metformin tablets.

## Introduction

Diabetes mellitus (DM) is a chronic disease in which the body exhibits compromised capability to produce or respond to insulin, leading to abnormal metabolism of carbohydrates and elevated levels of blood glucose. DM patients have long-term high blood glucose levels, which may cause chronic damage and dysfunction of various tissues/organs, including the eyes, kidneys, heart, blood vessels, and nerves (1). According to data released from the International Diabetes Federation (IDF) (2), the total number of DM patients aged 20-79 in the world reached 382 million in 2013, accounting for 8.3% of this age group. It was estimated that by 2035, these two figures will increase to 592 million and 10.1%, respectively (2). The World Health Organization (WHO) also predicted that by 2025, the number of DM patients in the world would exceed 300 million (3). DM has therefore become one of the three major chronic non-communicable diseases threatening human health worldwide. One type of DM is type 2 DM (T2DM), which is adult onset and the most common DM, accounting for 90% of all DM cases (4). T2DM is a progressive disease, and the principle of prevention and treatment for T2DM is to adopt strict blood glucose control strategies for newly diagnosed and early T2DM patients to prevent and delay the occurrence of diabetic complications (5, 6). For patients with long-term T2DM and existing complications, it is critical to adopt individualized blood sugar control strategies to control the progression of complications and reduce mortality (7).

It has been well established that metformin, a biguanide anti-hyperglycemic drug, which suppresses intestinal glucose absorption, reduces hepatic glucose production, enhances insulin sensitivity (8), can effectively reduce the blood glucose levels. Compared with sulfonylureas, another category of anti-hyperglycemic drug, metformin can significantly reduce the risk of chronic complications of T2DM patients (9). Hence, metformin has been extensively used in clinic as the first-line medication for T2DM treatment. However, previously, a meta-analysis showed that metformin had the worst medication compliance, with 30% of patients having failure in taking medication as required. That mega-analysis also suggested that poor medication compliance may be related to adverse drug reactions (ADRs) involving gastrointestinal system, central nervous system, cardiovascular system, and urinary system, and respiratory system caused by the metformin administration. Another study also suggested that poor compliance with medications for T2DM was linked to drug-associated adverse outcomes, including increased incidence of complications and increased overall treatment costs (10). Thus, it is important to reform the manufacturing procedure to generate different forms of metformin so that it may reduce the incidence and severity of ADRs while maintaining the therapeutic efficacy. Currently, metformin hydrochloride has two forms in clinical practice: ordinary immediate-release tablets and sustained-release (SR) tablets. The metformin SR tablet is an osmotic pump type of metformin hydrochloride SR tablet produced by the company Dulening, which uses a manufacturing process different from that of the matrix type metformin hydrochloride SR tablet. While previous studies revealed that the osmotic pump SR tablets had some advantages over metformin tablets and matrix SR tablets (11, 12), their safety and efficacy in treatment of T2DM have not been systemically compared.

In the present study, we adopted a randomized, open, positive drug parallel controlled clinical trial method to further evaluate and compare the efficacy and safety of Dulening metformin SR tablet II produced by Dulening with those of the original metformin tablets produced by Glucophage in T2DM treatment.

## Materials and Methods

### Research Design

This research adopted a randomized, open, positive drug parallel controlled multi-center clinical trial design. This study protocol was approved by the Ethics Committee of each involved institute and all participants signed the informed consent.

### Patient Selection

Patients who met all the following criteria were included in this study: (1) T2DM diagnosed based on the 1999 WHO diagnostic criteria; (2) no treatment with any blood glucose-lowering drugs after the initial clinical diagnosis, and treatment with metformin as proposed by doctors; (3) a serum glycated hemoglobin (HbA1c) level ≥7.5% but ≤ 11.0%; (4) a body mass index (BMI) ≥19 but ≤ 42; (5) an age between 18 and 75 years old; and 6) understanding of the procedures and methods of this study, willingness to strictly abide by the clinical trial protocol.

**Figure 1:**
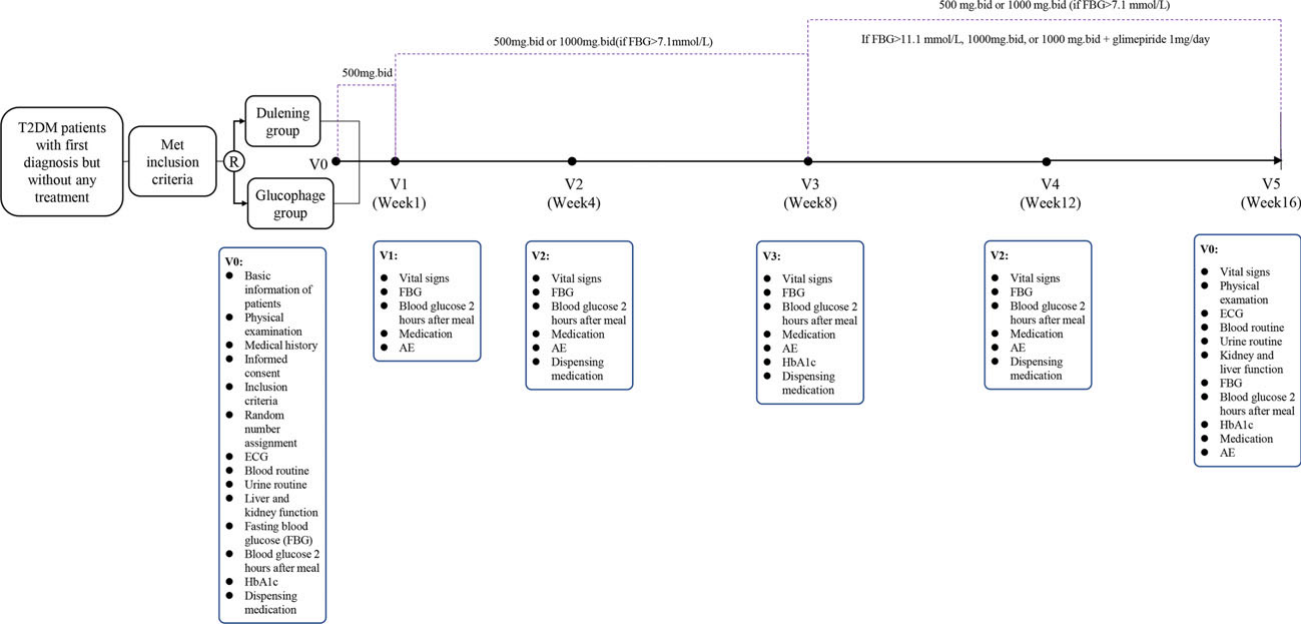
Flow chart showing patient selection process.

Patients who had one of the following characteristics were excluded from this study: (1) other diseases that needed to take medications that affected glucose metabolism; (2) pregnancy or preparation for pregnancy recently, or lactation; (3) hypertension that was difficult to control, i.e. the average systolic blood pressure ≥160 mmHg and diastolic blood pressure ≥100 mmHg, which were measured three consecutive times after antihypertensive treatment(s); (4) decompensated heart failure (NYHA classification III and IV), unstable angina, stroke or transient ischemic attack, myocardial infarction, arrhythmia, coronary artery bypass grafting or percutaneous coronary intervention, all of which occurred within 6 months before the initiation of randomization; (5) severe chronic gastrointestinal diseases before randomization or treatments that may affect drug absorption, such as gastrointestinal surgery; (6) abnormal liver and kidney function: serum levels of alanine aminotransferase (ALT) or aspartate aminotransferase (AST) were 3 times the upper limit of the normal level, and blood creatinine (Cr) was greater than the upper limit of the normal level; (7) mental or neurological diseases, unwillingness to communicate or language barriers, inability to fully understand and cooperate; (8) poor test compliance, i.e. those who were not resident in the region with unstable living and working environments; (9) alcoholism: drinking more than 50g daily; (10) participation in other clinical trials within 3 months before the initiation of randomization; and (11) any other factors that may affect the evaluation of this study as judged by the researchers. **Figure 1** shows the flowchart of patient selection process.

Drinking levels in this study were defined as follows: never drinking, defined as no white wine drinking at all; casual drinking, defined as drinking white wine once a week, each time <100g; and regular drinking, defined as drinking white wine weekly, each time ≥100g. Both casual and regular drinking belong to the drinking category. Obesity was defined as a BMI ≥ 28.0, overweight was defined as a 24 ≤ BMI ≤ 27.9, normal weight was defined as an 18.5 ≤ BMI ≤ 23.9, and underweight was defined as a BMI ≤ 18.4 (13).

### Medications

Patients took either Dulening or Glucophage 0.5g with meals twice a day. After 1 week of treatment, if fasting blood glucose (FBG) >7.1 mmol/L and this dosage of drug was tolerable, the dose was increased to 2000 mg per day, twice a day, 1.0g each time for 16 weeks as continuous medication. After the test dose reached the patient’s maximum tolerable dose or 2000 mg/day, if the patient’s HbA1c value at the 8th week was still 1.5% lower than the baseline or the FBG was still greater than 11.1 mmol/L, the investigator then decided whether combined medication was needed based on each individual patient’s exercise and diet control and medication situation. The additional anti-hyperglycemic drug was glimepiride provided by the sponsor, and the dosage was 1 mg once a day.

### Evaluation Indicators

The change in the serum HbA1c level after 16 weeks of treatment relative to its baseline level was used as the primary evaluation indicator of treatment efficacy, and the HbA1c compliance rate, changes in FBG and body weight were used as secondary evaluation indicators of treatment efficacy. ADRs, including adverse reactions of gastrointestinal system, central nervous system, cardiovascular system, and urinary system, and respiratory system, were used as safety indicators. *However, the vast majority of these adverse events were mild, and no special treatments were administered.*

### Sample Size Estimation

According to previous data and related literature, metformin monotherapy was estimated to reduce glycated hemoglobin by 0.9%-2.0%, and the US FDA recommends the HbA1c non-inferiority threshold of 0.3% or 0.4%. We assumed that the experimental drug and the control had comparable effects on lowering the HbA1c levels (δ=0%), and that the non-inferiority threshold was Δ=0.4%. According to relevant literature data, the combined standard deviation of the HbA1c difference between the two groups before and after treatment was s=1.38%. When α= 0.05 and the test power 1-β=0.9, according to the two-group equal sample size (1:1) designed by non-inferiority, the total sample size required for this clinical trial was at least 508 cases (in order to facilitate randomization, the integer number was 520). The test group and the control group each had 260 cases. Taking into account the 20% dropout rate, the total sample size was at least 624 cases. In addition, considering the characteristics of this study, HbA1c might not always meet the standard during the treatment and observation period and the need for combination medication and other factors, the total sample size of this study was determined to be 1000 cases, with either group having 500 cases. This study completed the observation of the planned sample size 1000 cases, and the number of analyzed cases accounted for 80% of the planned sample size.

### Statistical Analysis

All analyses were performed using the statistical software SAS9.3. One-sample Kolmogorov-Smirnov test was used to determine the normal distribution of data. Quantitative variables are expressed as mean±standard deviation (SD), median or quartiles (Q) and compared by student’s t test or analysis of variance (ANOVA) or Wilcoxon rank sum test as applicable. Categorical variables are expressed as a percentage (%), and compared with χ2 test. A difference was considered significant when a p value was less than 0.05.

## Results

### Comparison of Demographic and Basal Clinical Characteristics of Patients Between Dulening and Glucophage groups

Initially, a total of 1,000 patients with T2DM in 40 clinical centers who met the criteria for inclusion were randomly assigned to the Dulening and Glucophage groups. Among these patients, 853 (85.3%) completed all visits, and 147 (14.7%) dropped out during the study period, of whom 75 (15.0%) were in the Dulening group and 72 (14.4%) were in the Glucophage group. There was no statistically significant difference in the drop-off rate between these two groups (χ2 = 0.0718, P=0.7888). Eventually, a total of 886 cases were recruited, the Dulening group, n=446; and the Glucophage group, n=440), There were no statistically significant differences in demographic characteristics, diet and exercise status, and basal clinical characteristics between these two groups (**Tables 1**–**4**).

**Table 1:** Comparison of demographic characteristics of patients between Dulening and Glucophage groups.

Parameters	Dulening (n = 446)	Glucophage (n = 440)	t value	P value
Age (years)				
Mean ± SD	52.77 ± 10.28	53.10 ± 10.65	Z=0.5948	0.5519
Med (Q1,Q3)	53.75 (45.60,60.60)	54.25 (46.15,60.80)		
Min-Max	21.7-75.8	19.9-74.8		
Gender				
Male n (%)	257 (57.62)	268 (60.91)	*χ^2^ = *0.9904	0.3196
Female n (%)	189 (42.38)	172 (39.09)		
Weight (Kg)				
Mean ± SD	73.81 ± 13.14	74.08 ± 12.51	Z=0.5723	0.5671
Med (Q1,Q3)	73.00 (65.00,80.90)	72.75 (65.00,81.00)		
Min-Max	42.00-134.20	43.00-145.00		
Height (cm)				
Mean ± SD	166.53 ± 7.86	167.15 ± 7.85	Z=1.2906	0.1968
Median (Q1,Q3)	167.00 (160.00,172.00)	168.00 (160.00,173.00)		
Min-Max	149.00-185.00	149.00-192.00		
BMI				
Thin n (%)	1 (0.22)	1 (0.23)	*KW-χ^2^ = *0.0172	0.8956
Normal n (%)	110 (24.66)	101 (22.95)		
Overweight n (%)	201 (45.07)	210 (47.73)		
Obesity n (%)	134 (30.04)	128 (29.09)		
Nationality				
Han n (%)	431 (96.64)	428 (97.27)	*χ^2^ = *0.3032	0.5819
Others n (%)	15 (3.36)	12 (2.73)		
Residency				
City n (%)	333 (74.66)	329 (74.77)	*χ^2^ = *0.0014	0.9702
Countryside n (%)	113 (25.34)	111 (25.23)		
Education				
Primary school or below n (%)	44 (9.87)	39 (8.86)	Fisher	0.5390
Middle school n (%)	142 (31.84)	139 (31.59)		
High school n (%)	108 (24.22)	127 (28.86)		
Associate or Bachelor degree n (%)	148 (33.18)	130 (29.55)		
Master degree or above n (%)	4 (0.90)	5 (1.14)		

**Table 2:** Comparison of food customs and exercise status between the two groups.

Parameters	Dulening (n = 446)	Glucophage (n = 440)	t value	P value
Drinking				
Never n (%)	223 (50.00)	204 (46.36)	*χ^2^ = *1.3116	0.5190
Casual n (%)	199 (44.62)	208 (47.27)		
Regular n (%)	24 (5.38)	28 (6.36)		
Daily alcohol consumption (g)				
Mean ± SD	24.02 ± 15.64	25.09 ± 12.92	Z=-0.6241	0.5325
Med (Q1,Q3)	20.00 (10.00,40.00)	25.00 (17.50,30.00)		
Min-Max	1.5-50	5-50		
Dietary situation				
Light diet n (%)	72 (16.14)	64 (14.55)	*χ^2^ = *1.1395	0.5657
Moderate diet n (%)	312 (69.96)	322 (73.18)		
Sweeter diet n (%)	62 (13.90)	54 (12.27)		
Eating habits				
Mainly meat dishes n (%)	57 (12.78)	65 (14.77)	*χ^2^ = *0.8788	0.6444
Mainly vegetable dishes n (%)	64 (14.35)	58 (13.18)		
Mixed meat and vegetable n (%)	325 (72.87)	317 (72.05)		
Exercise				
No n (%)	170 (38.12)	176 (40.00)	*χ^2^ = *0.3938	0.8213
1-3 days/week n (%)	141 (31.61)	132 (30.00)		
>3 days/week n (%)	135 (30.27)	132 (30.00)		

**Table 3:** Comparison of basal characteristics of T2DM.

Parameters	Dulening (n = 446)	Glucophage (n = 440)	t value	*P* value
DM diagnosis				
First diagnosis n (%)	322 (72.20)	326 (74.09)	*χ^2^ = *0.4042	0.5249
Previous diagnosis n (%)	124 (27.80)	114 (25.91)		
Diabetic course (montu)				
Mean ± SD	29.50 ± 31.75	26.53 ± 34.72	Z=-1.3711	0.1703
Med (Q1,Q3)	17.30 (10.45,40.80)	13.60 (7.20,33.90)		
Min-Max	0.1-211.6	0.2-183.9		
Diabetic neuropathy				
Yes n (%)	442 (99.10)	433 (98.41)	*χ^2^ = *0.8702	0.3509
No n (%)	4 (0.90)	7 (1.59)		
Diabetic retinopathy				
Yes n (%)	442 (99.10)	432 (98.18)	*χ^2^ = *1.4072	0.2355
No n (%)	4 (0.90)	8 (1.82)		
Diabetic nephropathy				
Yes n (%)	442 (99.10)	434 (98.64)	Fisher	0.5440
No n (%)	4 (0.90)	6 (1.36)		
Comorbidity				
Yes n (%)	225 (50.45)	240 (54.55)	*χ^2^ = *1.4908	0.2221
No n (%)	221 (49.55)	200 (45.45)		

**Table 4:** Comparison of efficacy-related parameters of patients between two groups.

Parameters	Dulening (n = 446)	Glucophage (n = 440)	t value	*P* value
HbAc1 (%)				
Mean ± SD	8.63 ± 1.01	8.59 ± 0.94	Z=-0.1075	0.9144
Med (Q1,Q3)	8.40 (7.80,9.30)	8.30 (7.80,9.20)		
Min-Max	7.5-11	7.5-11		
HbAc1 composition				
7.5%-	144 (32.29)	137 (31.14)	*χ^2^ = *5.6466	0.4639
8.0%-	91 (20.40)	108 (24.55)		
8.5%-	73 (16.37)	64 (14.55)		
9.0%-	46 (10.31)	47 (10.68)		
9.5%-	31 (6.95)	33 (7.50)		
10.0%-	22 (4.93)	26 (5.91)		
10.5%-	39 (8.74)	25 (5.68)		
FBG (mmol/L)				
Mean ± SD	9.86 ± 2.39	9.95 ± 2.59	Z=-0.0293	0.9766
Med (Q1,Q3)	9.40 (8.20,10.90)	9.30 (8.20,11.10)		
Min-Max	4.3,22	4.7,21.2		

### Comparison of Treatment Efficacy Between Two Groups

General data showing the changes in clinical parameters such as liver and kidney function, fasting insulin levels, LDL, HDL, and triglyceride before and posttreatment of subjects in two groups are summarized in Supplemental tables 1 and 2. Specifically, the serum HbA1c level in the Dulening group dropped from 8.63% at baseline to 7.40% in the 8^th^ week and to 6.94% in the 16^th^ week, respectively, and the serum HbA1c level in the Glucophage group dropped from 8.59% at baseline to 7.25% in the 8th week and 6.80% in the 16th week, respectively. The average reduction in the serum HbA1c levels in these two groups was between 1.6-1.8%, and no significant difference in the reduction of serum HbA1c was found between these two groups (P =0.2799, **Table 5**).

**Table 5:** Analysis of changes in HbAc1 levels at different time points in patients of two groups.

Time	Dulening (n = 446)	Glucophage (n = 440)	t value	*P* value
Baseline	8.63 ± 1.01	8.59 ± 0.94	Z=-0.1075	0.9144
8^th^ week	7.40 ± 1.06	7.25 ± 1.00	Z=-2.0137	0.0440
16^th^ week	6.94 ± 1.01	6.80 ± 1.01	Z=-2.8223	0.0048
Δ between baseline and 8^th^ week	1.23 ± 1.07	1.34 ± 1.09	Z=1.4117	0.1580
Δ between baseline and 16^th^ week	1.69 ± 1.24	1.78 ± 1.24	Z=1.7587	0.0786

Data are expressed as mean ± SD. ANOVA: different time points: F=1139.39, P<0.0001; time and group interaction: F=1.27, P=0.2799.

The FBG decreased from 9.86 mmol/L at the beginning of the treatment to 7.53 mmol/L at the end of the study in the Dulenin group, and from 9.95 mmol/L at the beginning of the treatment to 7.25 mmol/L at the end of the study in the Glucophage group. Both treatments significantly decreased the FBG (p<0.0001, **Table 6**), but there was no significant difference in the reduction of FBG between these two groups (p>0.05, **Table 6**).

**Table 6:** Comparison of changes in FBG of patients at different time points between two groups.

Group	Baseline	1^st^ week	4^th^ week	8^th^ week	12^th^ week	16^th^ week
Dulening (n=446)	9.86 ± 2.39	8.58 ± 2.04	8.00 ± 1.64	7.73 ± 1.54	7.58 ± 1.56	7.53 ± 1.53
Glucophage (n=440)	9.95 ± 2.59	8.59 ± 1.95	7.94 ± 1.67	7.69 ± 1.46	7.41 ± 1.46	7.25 ± 1.40

Data are expressed as mean ± SD. ANOVA: different time points: F=422.85, P<0.0001; time and group interaction: F=1.62, P=0.1508.

During the study period, individual patient in the Dulening and Glucophage groups lost 1.3kg and 1.5kg on average, respectively, and the weight loss before and after treatment was significant for either group (p<0.0001, **Table 7**). However, the difference in weight loss between these two groups was not statistically significant (p=0.1421, **Table 7**).

**Table 7:** Comparison of changes in body weight of patients at different time points between two groups.

Group	Baseline	1^st^ week	4^th^ week	8^th^ week	12^th^ week	16^th^ week
Dulening (n=446)	73.8 ± 13.1	73.7 ± 13.1	73.3 ± 12.9	73.0 ± 12.9	72.8 ± 12.8	72.5 ± 12.8
Glucophage (n=440)	74.1 ± 12.5	73.9 ± 12.5	73.6 ± 12.4	73.1 ± 12.2	72.8 ± 12.2	72.6 ± 12.1

Data are expressed as mean ± SD. ANOVA: different time points: F=174.28, P<0.0001; time and group interaction: F=1.65, P=0.1421.

### Comparison of Safety Between Two Groups

**Figure 2:**
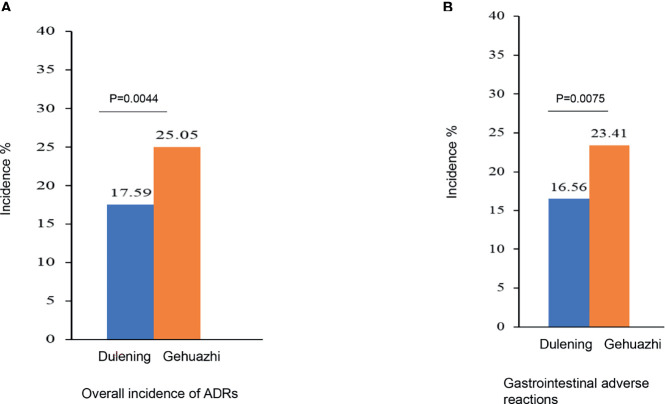
The Dulening group had a significantly lower incidence of ADRs **(A)** and lower incidence of gastrointestinal ADR **(B)** than the Glucophage group.

The Dulening group had a significantly lower incidence of ADRs compared with the Glucophage group (17.59% *vs*. 25.05%. χ2 = 8.1069, P =0.0044. **Figure 2A**). The primary ADR was on the digestive system, and the Dulening group had a significantly lower incidence of gastrointestinal adverse reaction than the Glucophage group (16.56% *vs*. 23.41%. X^2^ = 7.1490, P=0.0075. **Figure 2B**).

## Discussion

Metformin, as the first-line drug of choice for oral medication for T2DM patients, is recommended by almost all DM treatment guidelines, and its therapeutic efficacy has been well documented. However, different forms of metformin have different efficacies in T2DM treatment. The present study investigated the efficacy and safety of two different forms of metformin, the Dulening SR tablets and the Glucophage original metformin tablets, in treatment of T2DM patients. We found that both forms of metformin had comparable efficacy in this treatment, but the SR tablets had significantly lower ADRs, primarily the gastrointestinal adverse reactions, than the original metformin form. Therefore, we conclude that the SR tablets produced by Dulening are safer than the original tablet produced by Glucophage.

Dulening metformin SR tablets were produced using a manufacturing process that was different from the original metformin form produced by Glucophage. Previous studies suggested that 1) the osmotic pump used by Dulening released metformin at a uniform rate, with zero-level release *in vitro*, 2) the amount of released metformin was able to maintain the blood glucose-lowering effect, 3) the action time lasted long, and 4) the incidence of adverse gastrointestinal reactions was low (11, 12). To further evaluate the efficacy and safety of the Dulening metformin SR tablets in T2DM treatment, in the present study, we compared it with Glucophage metformin tablets in a randomized, open, and positive drug control trial. This clinical trial design is regarded as a reliable clinical trial design in the field (14). We used FBG and HbA1c as efficacy indicators for T2DM treatment, and these two indicators are well recognized for their reliability in clinic to monitor DM treatment efficacy (15). We treated T2DM patients for 16 weeks, and found that both forms of metformin effectively reduced FBG and HbA1c with comparable efficacy. In addition, these two forms of metformin also significantly reduced the body weight of patients in both groups in an equivalent efficiency. While highly elevated blood glucose may promote degradation of muscles and thus lead to weight loss, weight loss is also regarded as a result of appropriate management of T2DM (16). In this study, T2DM patients did not have very high FBG even at baseline, so we believe that the weight loss observed in these patients was the favorable outcome of treatment in this study. Hence, we conclude that the Dulening metformin SR tablets and Glucophage metformin tablets exhibit comparable efficacy in treatment of T2DM patients.

During T2DM treatment, whether or not to adhere to the medication protocol as prescribed is an important factor in determining the effectiveness of the treatment. Fluctuations in the serum drug levels will lead to fluctuation in blood glucose levels, eventually resulting in reduced therapeutic efficacy and increased incidence of DM complications (17). As mentioned above, ADRs are the primary cause for T2DM patients who were administered with metformin and had poor medication compliance. Previously, Blond et al. performed a study to examine the tolerance of ADRs caused by metformin extended-release tablets, and found that the ADR of the digestive tract was 11.71%, which was lower than the 26.34% caused by the ordinary tablets of metformin (10). However, Garber et al. showed that that the incidence of gastrointestinal ADRs was 28%, probably because the initial dose of metformin was large, i.e. 1000 mg/d (18). In the present study, we examined the safety of these two forms of metformin first based on the overall incidence of ADRs and then on the incidence of gastrointestinal ADR, the latter of which is the primary ADR caused by metformin administration (19) (data not shown). We found that the Duleming metformin SR tablets had a significantly lower overall incidence of ADRs as well as the lower incidence of gastrointestinal ADRs than the Glucophage metformin tablets. Our observations were in line with Blond’s findings (10). Hence, we argue that the SR tablets of metformin produced by Dulening had better safety than the original form of metformin produced by Glucophage. The reasons accounting for the better safety of SR tablets are likely attributable to the unique membrane-controlled slow-release technology, which can ensure that the drug release rate is constant after the drug enters the body, subsequently reducing the irritation to the gastrointestinal tract.

The limitations of this study should be noted. First, the experimental design did not take into account the impact of the disease on the quality of life of patients. Second, the use of the general EQ-5D questionnaire might not have sufficient sensitivity for measurement of the quality of life of patients. Third, we failed to obtain circulating levels of GLP-1 and fasting insulin of 60 blood samples due to technical issues, which might have a certain degree of impact on our findings. Therefore, future prospective studies with large cohorts should be performed to corroborate our conclusions.

In summary, our study demonstrates that the Dulening metformin SR tablets (II) exhibit comparable efficacy in T2DM treatment compared with the original form of metformin but have better safety, as evidenced by reduced overall incidence of ADRs and the incidence of gastrointestinal ADR, while they possess similar therapeutic efficacy. Thus, the SR tablets of metformin produced by Dulening should hold promise in clinical practice to treat T2DM patients in the future.

## Data Availability Statement

The original contributions presented in the study are included in the article/**Supplementary Material**. Further inquiries can be directed to the corresponding author.

## Ethics Statement

This study protocol was approved by the Ethic Committee of each involved institute and all participants signed the informed consent. The patients/participants provided their written informed consent to participate in this study.

## Author Contributions

L-xG, G-eL and LC designed the study and wrote the manuscript. H-fW, JG, X-lZ, B-hD, D-zW, WZ and KW collected the data. W-sT, QC, Q-zL, JY, QZ, P-fX and M-xL analyzed the data. All authors contributed to the article and approved the submitted version.

## Conflict of Interest

The authors declare that the research was conducted in the absence of any commercial or financial relationships that could be construed as a potential conflict of interest.

## Publisher’s Note

All claims expressed in this article are solely those of the authors and do not necessarily represent those of their affiliated organizations, or those of the publisher, the editors and the reviewers. Any product that may be evaluated in this article, or claim that may be made by its manufacturer, is not guaranteed or endorsed by the publisher.

## References

[1] Papatheodorou K Banach M Bekiari E Rizzo M Edmonds M. Complications of Diabetes 2017. J Diabetes Res (2018) 2018:3086167. doi: 10.1155/2018/3086167 29713648 PMC5866895

[2] Aguiree F Brown A Cho NH Dahlquist G Dodd S Dunning T. IDF Diabetes Atlas: Sixth Edition. Brussels, Belgium: International Diabetes Federation (2013).

[3] Organization PAH. The World Health Report 1998: Life in the 21st Century A Vision for All. Geneva Switzerland: Who (1998) p. 391–2.

[4] Zheng Y Ley SH Hu FB. Global Aetiology and Epidemiology of Type 2 Diabetes Mellitus and Its Complications. Nat Rev Endocrinol (2018) 14:88–98. doi: 10.1038/nrendo.2017.151 29219149

[5] Stumvoll M Goldstein BJ van Haeften TW. Type 2 Diabetes: Principles of Pathogenesis and Therapy. Lancet (2005) 365:1333–46. doi: 10.1016/s0140-6736(05)61032-x 15823385

[6] Asif M. The Prevention and Control the Type-2 Diabetes by Changing Lifestyle and Dietary Pattern. J Educ Health Promot (2014) 3:1. doi: 10.4103/2277-9531.127541 24741641 PMC3977406

[7] Chinese DS. Chinese Type 2 Diabetes Prevention and Treatment Guidelines (2013 Edition). Chin J Endocrinol Metab (2014) 30:893–942. doi: 10.3760/cma.j.issn.1000-6699.2014.10.020

[8] Pernicova I Korbonits M. Metformin–Mode of Action and Clinical Implications for Diabetes and Cancer. Nat Rev Endocrinol (2014) 10:143–56. doi: 10.1038/nrendo.2013.256 24393785

[9] Fujioka K Brazg RL Raz I Bruce S Joyal S Swanink R. Efficacy, Dose-Response Relationship and Safety of Once-Daily Extended-Release Metformin (Glucophage XR) in Type 2 Diabetic Patients With Inadequate Glycaemic Control Despite Prior Treatment With Diet and Exercise: Results From Two Double-Blind, Placebo-Controlled Studies. Diabetes Obes Metab (2005) 7:28–39. doi: 10.1111/j.1463-1326.2004.00369.x 15642073

[10] Blonde L Dailey GE Jabbour SA Reasner CA Mills DJ. Gastrointestinal Tolerability of Extended-Release Metformin Tablets Compared to Immediate-Release Metformin Tablets: Results of a Retrospective Cohort Study. Curr Med Res Opin (2004) 20:565–72. doi: 10.1185/030079904125003278 15119994

[11] Yang J Qi YR. Correlation Between the *In Vitro* Release and *In Vivo* Absorption of Metformin Hydrochloride Sustained-Release Tablets. Chin Pharm J (2011) 46:1727–9. doi: 10.1631/jzus.B1000135

[12] Zhang ZY Mei Y Yang L Luo L Cai M Xu XM. Analysis of Clinical Efficacy and Safety of Osmotic Pump and Matrix Type Metformin Sustained-Release Tablets in the Treatment of Type 2 Diabetes. J Baotou Med Coll (2016) 000:49–50. doi: CNKI:SUN:BTYX.0.2016-02-030

[13] Komaroff M. For Researchers on Obesity: Historical Review of Extra Body Weight Definitions. J Obes (2016) 2016:2460285. doi: 10.1155/2016/2460285 27313875 PMC4904092

[14] Naci H Davis C Savović J Higgins JPT Sterne JAC Gyawali B. Design Characteristics, Risk of Bias, and Reporting of Randomised Controlled Trials Supporting Approvals of Cancer Drugs by European Medicines Agency, 2014-16: Cross Sectional Analysis. BMJ (2019) 366:l5221. doi: 10.1136/bmj.l5221 31533922 PMC6749182

[15] Mandal AK Hiebert L. Diagnosis and Management of Diabetes and the Relationship of Dglucose to Kidney Function. Curr Diabetes Rev (2015) 11:116–21. doi: 10.2174/1573399811666150302111453 PMC4443797 25732030

[16] Wilding JP. The Importance of Weight Management in Type 2 Diabetes Mellitus. Int J Clin Pract (2014) 68:682–91. doi: 10.1111/ijcp.12384 PMC4238418 24548654

[17] Dandona P. Minimizing Glycemic Fluctuations in Patients With Type 2 Diabetes: Approaches and Importance. Diabetes Technol Ther (2017) 19:498–506. doi: 10.1089/dia.2016.0372 28771387 PMC5647495

[18] Garber AJ Duncan TG Goodman AM Mills DJ Rohlf JL. Efficacy of Metformin in Type II Diabetes: Results of a Double-Blind, Placebo-Controlled, Dose-Response Trial. Am J Med (1997) 103:491–7. doi: 10.1016/s0002-9343(97)00254-4 9428832

[19] Fatima M Sadeeqa S Nazir S. Metformin and its Gastrointestinal Problems: A Review. Biomed Res (2018) 29:2285–9. doi: 10.4066/biomedicalresearch.40-18-526

