# Supplementary material for: Comparison of Clinical Efficacy and Safety of Metformin Sustained-Release Tablet (II) (Dulening) and Metformin Tablet (Glucophage) in Treatment of Type 2 Diabetes Mellitus
Source: Front Endocrinol (Lausanne). 2021 Sep 30;12:712200. doi: 10.3389/fendo.2021.712200 (PMC8515195; doi:10.3389/fendo.2021.712200)
Supplement: Supplementary file 1 [file Table_1.docx]

**Supplemental table 1. Changes of laboratory indicators-liver, kidney, and blood lipids**

| Posttreatment | Pretreatment n (%) | | | | | | | |
| --- | --- | --- | --- | --- | --- | --- | --- | --- |
|  | Dulening (n=489) | | | | Glucophage (n=487) | | | |
|  | Normal | Abnormal- | Abnormal+ | Not examined | Normal | Abnormal- | Abnormal+ | Not examined |
| **Aspartate aminotransferase** |  |  |  |  |  |  |  |  |
| Normal | 317(76.02) | 30(68.18) | 9(50.00) | 4(40.00) | 333(80.05) | 17(45.95) | 12(52.17) | 3(27.27) |
| Abnormal- | 19(4.56) | 8(18.18) | 0(0.00) | 2(20.00) | 18(4.33) | 14(37.84) | 0(0.00) | 1(9.09) |
| Abnormal+ | 6(1.44) | 1(2.27) | 4(22.22) | 0(0.00) | 1(0.24) | 0(0.00) | 7(30.43) | 0(0.00) |
| Not examined | 75(17.99) | 5(11.36) | 5(27.78) | 4(40.00) | 64(15.38) | 6(16.22) | 4(17.39) | 7(63.64) |
| **Alanine aminotransferase** |  |  |  |  |  |  |  |  |
| Normal | 309(80.05) | 41(63.08) | 18(54.55) | 4(80.00) | 328(82.41) | 34(64.15) | 14(45.16) | 3(60.00) |
| Abnormal- | 14(3.63) | 15(23.08) | 4(12.12) | 1(20.00) | 15(3.77) | 12(22.64) | 4(12.90) | 0(0.00) |
| Abnormal+ | 7(1.81) | 2(3.08) | 6(18.18) | 0(0.00) | 5(1.26) | 1(1.89) | 10(32.26) | 0(0.00) |
| Not examined | 56(14.51) | 7(10.77) | 5(15.15) | 0(0.00) | 50(12.56) | 6(11.32) | 3(9.68) | 2(40.00) |
| **γ-glutamyl transpeptidase** |  |  |  |  |  |  |  |  |
| Normal | 294(80.77) | 28(47.46) | 9(28.13) | 20(58.82) | 291(82.67) | 39(60.00) | 15(34.88) | 14(51.85) |
| Abnormal- | 20(5.49) | 21(35.59) | 2(6.25) | 2(5.88) | 12(3.41) | 17(26.15) | 4(9.30) | 3(11.11) |
| Abnormal+ | 3(0.82) | 2(3.39) | 10(31.25) | 1(2.94) | 2(0.57) | 3(4.62) | 17(39.53) | 1(3.70) |
| Not examined | 47(12.91) | 8(13.56) | 11(34.38) | 11(32.35) | 47(13.35) | 6(9.23) | 7(16.28) | 9(33.33) |
| **Alkaline phosphatase** |  |  |  |  |  |  |  |  |
| Normal | 346(83.17) | 21(63.64) | 3(50.00) | 22(64.71) | 356(84.76) | 21(61.76) | 3(60.00) | 17(60.71) |
| Abnormal- | 9(2.16) | 11(33.33) | 2(33.33) | 0(0.00) | 11(2.62) | 5(14.71) | 0(0.00) | 2(7.14) |
| Abnormal+ | 0(0.00) | 0(0.00) | 0(0.00) | 0(0.00) | 0(0.00) | 3(8.82) | 2(40.00) | 0(0.00) |
| Not examined | 61(14.66) | 1(3.03) | 1(16.67) | 12(35.29) | 53(12.62) | 5(14.71) | 0(0.00) | 9(32.14) |
| **Urea nitrogen** |  |  |  |  |  |  |  |  |
| Normal | 377(82.31) | 20(86.96) | 1(50.00) | 6(100.00) | 391(85.00) | 9(60.00) | 0(0.00) | 8(66.67) |
| Abnormal- | 16(3.49) | 1(4.35) | 0(0.00) | 0(0.00) | 11(2.39) | 4(26.67) | 0(0.00) | 2(16.67) |
| Abnormal+ | 0(0.00) | 0(0.00) | 0(0.00) | 0(0.00) | 0(0.00) | 0(0.00) | 0(0.00) | 0(0.00) |
| Not examined | 65(14.19) | 2(8.70) | 1(50.00) | 0(0.00) | 58(12.61) | 2(13.33) | 0(0.00) | 2(16.67) |
| **Creatinine** |  |  |  |  |  |  |  |  |
| Normal | 340(79.07) | 23(43.40) | 0(0.00) | 4(80.00) | 351(82.20) | 23(46.94) | 0(0.00) | 6(60.00) |
| Abnormal- | 29(6.74) | 23(43.40) | 0(0.00) | 1(20.00) | 25(5.85) | 17(34.69) | 1(100.00) | 2(20.00) |
| Abnormal+ | 1(0.23) | 0(0.00) | 0(0.00) | 0(0.00) | 0(0.00) | 0(0.00) | 0(0.00) | 0(0.00) |
| Not examined | 60(13.95) | 7(13.21) | 1(100.00) | 0(0.00) | 51(11.94) | 9(18.37) | 0(0.00) | 2(20.00) |
| **Total bilirubin** |  |  |  |  |  |  |  |  |
| Normal | 366(82.06) | 22(68.75) | 1(33.33) | 5(62.50) | 350(82.35) | 25(51.02) | 1(33.33) | 7(70.00) |
| Abnormal- | 13(2.91) | 7(21.88) | 0(0.00) | 1(12.50) | 17(4.00) | 18(36.73) | 2(66.67) | 1(10.00) |
| Abnormal+ | 1(0.22) | 1(3.13) | 2(66.67) | 0(0.00) | 2(0.47) | 1(2.04) | 0(0.00) | 0(0.00) |
| Not examined | 66(14.80) | 2(6.25) | 0(0.00) | 2(25.00) | 56(13.18) | 5(10.20) | 0(0.00) | 2(20.00) |
| **Total cholesterol** |  |  |  |  |  |  |  |  |
| Normal | 245(78.78) | 19(57.58) | 55(40.74) | 8(80.00) | 234(76.47) | 23(56.10) | 62(47.69) | 7(70.00) |
| Abnormal- | 19(6.11) | 11(33.33) | 7(5.19) | 0(0.00) | 19(6.21) | 12(29.27) | 14(10.77) | 0(0.00) |
| Abnormal+ | 5(1.61) | 0(0.00) | 46(34.07) | 0(0.00) | 10(3.27) | 3(7.32) | 37(28.46) | 0(0.00) |
| Not examined | 42(13.50) | 3(9.09) | 27(20.00) | 2(20.00) | 43(14.05) | 3(7.32) | 17(13.08) | 3(30.00) |
| **Triglycerides** |  |  |  |  |  |  |  |  |
| Normal | 172(67.72) | 24(46.15) | 51(29.14) | 5(62.50) | 157(68.26) | 28(42.42) | 62(34.25) | 2(20.00) |
| Abnormal- | 29(11.42) | 19(36.54) | 10(5.71) | 1(12.50) | 23(10.00) | 25(37.88) | 17(9.39) | 3(30.00) |
| Abnormal+ | 16(6.30) | 3(5.77) | 86(49.14) | 1(12.50) | 16(6.96) | 4(6.06) | 82(45.30) | 1(10.00) |
| Not examined | 37(14.57) | 6(11.54) | 28(16.00) | 1(12.50) | 34(14.78) | 9(13.64) | 20(11.05) | 4(40.00) |
| **High density lipoprotein** |  |  |  |  |  |  |  |  |
| Normal | 286(77.72) | 30(38.96) | 17(44.74) | 3(50.00) | 292(77.04) | 33(48.53) | 15(44.12) | 3(50.00) |
| Abnormal- | 20(5.43) | 37(48.05) | 6(15.79) | 0(0.00) | 29(7.65) | 24(35.29) | 5(14.71) | 1(16.67) |
| Abnormal+ | 4(1.09) | 4(5.19) | 10(26.32) | 1(16.67) | 8(2.11) | 0(0.00) | 9(26.47) | 0(0.00) |
| Not examined | 58(15.76) | 6(7.79) | 5(13.16) | 2(33.33) | 50(13.19) | 11(16.18) | 5(14.71) | 2(33.33) |
| **Low density lipoprotein** |  |  |  |  |  |  |  |  |
| Normal | 224(73.20) | 28(44.44) | 52(45.22) | 4(80.00) | 206(72.03) | 24(39.34) | 57(43.18) | 4(57.14) |
| Abnormal- | 25(8.17) | 22(34.92) | 5(4.35) | 0(0.00) | 27(9.44) | 24(39.34) | 21(15.91) | 1(14.29) |
| Abnormal+ | 11(3.59) | 4(6.35) | 41(35.65) | 0(0.00) | 15(5.24) | 3(4.92) | 37(28.03) | 0(0.00) |
| Not examined | 46(15.03) | 9(14.29) | 17(14.78) | 1(20.00) | 38(13.29) | 10(16.39) | 17(12.88) | 2(28.57) |

Note: % means the percentage of change after treatment based on the corresponding pretreatment parameter. Abnormal -: abnormal with no clinical significance; Abnormal +: abnormal with clinical significance.
