# Supplementary material for: Comparison of Clinical Efficacy and Safety of Metformin Sustained-Release Tablet (II) (Dulening) and Metformin Tablet (Glucophage) in Treatment of Type 2 Diabetes Mellitus
Source: Front Endocrinol (Lausanne). 2021 Sep 30;12:712200. doi: 10.3389/fendo.2021.712200 (PMC8515195; doi:10.3389/fendo.2021.712200)
Supplement: Supplementary file 2 [file Table_2.docx]

**Table 2. Changes in laboratory indicators-liver, kidney, and blood lipids**

| Indicators | Dulening (n=489) | Glucophage (n=487) | t value | *P* value |
| --- | --- | --- | --- | --- |
| **Urea nitrogen-baseline** | 5.14±1.43 | 5.33±2.91 | Z=0.2784 | 0.7807 |
| **Urea nitrogen-posttreatment** | 6.65±16.26 | 5.13±1.25 | Z=2.0209 | **0.0433*** |
| **Δ Urea nitrogen** | -1.54±16.29 | 0.25±3.16 | Z=-2.0569 | **0.0397*** |
| **Creatinine-baseline** | 64.77±16.69 | 64.09±16.37 | Z=-0.5955 | 0.5515 |
| **Creatinine-posttreatment** | 65.42±20.19 | 65.67±18.51 | Z=-0.3991 | 0.6899 |
| **Δ Creatinine** | -0.90±18.35 | -0.94±17.60 | Z=-0.1989 | 0.8423 |
| **Aspartate aminotransferase-baseline** | 24.64±11.04 | 24.98±12.57 | Z=0.0335 | 0.9733 |
| **Aspartate aminotransferase-posttreatment** | 22.73±10.17 | 22.82±17.50 | Z=1.4770 | 0.1397 |
| **ΔAspartate aminotransferase** | 1.91±11.38 | 1.93±17.03 | Z=-1.1840 | 0.2364 |
| **Alanine aminotransferase-baseline** | 31.89±20.05 | 31.24±20.80 | Z=-0.7266 | 0.4675 |
| **Alanine aminotransferase-posttreatment** | 27.12±18.26 | 26.40±16.97 | Z=0.7636 | 0.4451 |
| **ΔAlanine aminotransferase** | 4.62±19.52 | 4.95±18.02 | Z=-0.7060 | 0.4802 |
| **γ-glutamyl transpeptidase-baseline** | 41.43±41.76 | 42.87±35.94 | Z=-0.7572 | 0.4489 |
| **γ-glutamyl transpeptidase-posttreatment** | 34.08±26.38 | 37.36±51.88 | Z=0.2860 | 0.7749 |
| **Δγ-glutamyl transpeptidase** | 6.87±39.80 | 5.39±45.63 | Z=-1.7338 | 0.0829 |
| **Alkaline phosphatase-baseline** | 83.23±29.01 | 82.21±26.48 | Z=0.6144 | 0.5390 |
| **Alkaline phosphatase-posttreatment** | 74.92±26.48 | 73.33±22.38 | Z=0.6343 | 0.5259 |
| **ΔAlkaline phosphatase** | 8.08±20.25 | 7.41±22.32 | Z=-0.0144 | 0.9885 |
| **Total bilirubin-baseline** | 14.48±8.31 | 15.85±13.86 | Z=1.4055 | 0.1599 |
| **Total bilirubin-posttreatment** | 13.92±8.23 | 13.97±6.28 | Z=-0.0035 | 0.9972 |
| **ΔTotal bilirubin** | 0.62±10.71 | 2.19±13.86 | Z=-1.4644 | 0.1431 |
| **Total cholesterol-baseline** | 5.42±4.08 | 5.43±3.90 | Z=-0.8668 | 0.3861 |
| **Total cholesterol-posttreatment** | 4.96±3.00 | 4.78±1.06 | Z=0.5339 | 0.5934 |
| **ΔTotal cholesterol** | 0.47±5.18 | 0.67±4.14 | Z=-0.1882 | 0.8507 |
| **Triglyceride-baseline** | 2.32±1.87 | 3.29±17.40 | Z=0.9471 | 0.3436 |
| **Triglyceride-posttreatment** | 2.41±7.98 | 2.41±7.23 | Z=0.1432 | 0.8861 |
| **ΔTriglyceride** | -0.11±8.11 | 0.95±19.78 | Z=-1.2165 | 0.2238 |
| **HDL cholesterol-baseline** | 1.47±5.46 | 1.23±0.37 | Z=0.2045 | 0.8379 |
| **HDL cholesterol-posttreatment** | 1.21±0.28 | 1.20±0.25 | Z=0.0953 | 0.9240 |
| **Δ HDL cholesterol** | 0.30±5.89 | 0.03±0.36 | Z=-1.0600 | 0.2891 |
| **LDL cholesterol-baseline** | 3.12±0.87 | 3.14±0.93 | Z=0.3459 | 0.7294 |
| **LDL cholesterol-posttreatment** | 2.87±0.80 | 3.40±10.63 | Z=-0.2993 | 0.7647 |
| **ΔLDL cholesterol** | 0.24±0.87 | -0.26±10.72 | Z=-0.3790 | 0.7047 |
| **Fasting insulin-baseline** | 30.05±23.16 | 39.22±34.28 | Z=-0.9403 | 0.3470 |
| **Fasting insulin-posttreatment** | 26.61±12.23 | 28.25±1.91 | Z=-0.1205 | 0.9041 |
| **Δ** **Fasting insulin** | -4.98±8.16 | -0.25±2.60 | Z=1.8995 | 0.0575 |
| **Glycated hemoglobin-baseline** | 8.62±1.00 | 8.60±0.94 | Z=0.2444 | 0.8069 |
| **Glycated hemoglobin-posttreatment** | 7.08±1.10 | 6.99±1.15 | Z=-2.1213 | **0.0339*** |
| **ΔGlycated hemoglobin-baseline** | 1.54±1.27 | 1.62±1.29 | Z=1.3564 | 0.1750 |

Δ, the difference between posttreatment and baseline; HDL, high density lipid; LDL, low density lipid; *, p value indicates p<0.05.
